# A Bayesian Approach to Predict Blast-Induced Damage of High Rock Slope Using Vibration and Sonic Data

**DOI:** 10.3390/s21072473

**Published:** 2021-04-02

**Authors:** Pengchang Sun, Wenbo Lu, Haoran Hu, Yuzhu Zhang, Ming Chen, Peng Yan

**Affiliations:** 1 State Key Laboratory of Water Resources and Hydropower Engineering Science, Wuhan University, Wuhan 430072, China; whuchm@whu.edu.cn (M.C.); pyanwhu@whu.edu.cn (P.Y.); 2 Key Laboratory of Rock Mechanics in Hydraulic Structural Engineering of Ministry of Education, Wuhan University, Wuhan 430072, China; 3 Changjiang Institute of Survey, Planning, Design and Research, Wuhan 430010, China; hrhu@whu.edu.cn (H.H.); yzzhang@whu.edu.cn (Y.Z.)

**Keywords:** blast-induced damage, high rock slope, sonic test, blasting vibration, natural frequency, Bayesian linear regression

## Abstract

The blast-induced damage of a high rock slope is directly related to construction safety and the operation performance of the slope. Approaches currently used to measure and predict the blast-induced damage are time-consuming and costly. A Bayesian approach was proposed to predict the blast-induced damage of high rock slopes using vibration and sonic data. The relationship between the blast-induced damage and the natural frequency of the rock mass was firstly developed. Based on the developed relationship, specific procedures of the Bayesian approach were then illustrated. Finally, the proposed approach was used to predict the blast-induced damage of the rock slope at the Baihetan Hydropower Station. The results showed that the damage depth representing the blast-induced damage is proportional to the change in the natural frequency. The first step of the approach is establishing a predictive model by undertaking Bayesian linear regression, and the second step is predicting the damage depth for the next bench blasting by inputting the change rate in the natural frequency into the predictive model. Probabilities of predicted results being below corresponding observations are all above 0.85. The approach can make the best of observations and includes uncertainty in predicted results.

## 1. Introduction

Excavation of high rock slopes in many fields, such as transportation, hydraulic and hydropower, and mining engineering, usually involves blasting due to the high efficiency, reliable effectiveness, and low costs of blasting operations [1,2,3]. During excavation of rock slopes, blasting loads with high levels arisen from blasting operations close to contour surfaces usually trigger unavoidable damage in remaining rock masses, which impairs the integrity and strength of the remaining rock masses that support different major structures, such as dams and bridges [4,5]. The blast-induced damage could cause local or even complete failure of rock slopes, and thus heavy economic losses and even major catastrophes in an extensive region follow closely [6,7].

Measurement techniques currently used to detect the blast-induced damage of high rock slopes can be naturally divided into two categories, direct measurements and non-direct measurements [8]. As regards direct measurements, geometric and topological features of micro defects in rock masses are directly measured, and those measured features, such as the number, size, shape and position of the micro defects, are then used to quantitatively define the rock damage. According to the locations of the measured micro defects, the direct measurement is further subdivided into superficial topography measurements, which make use of diverse optical microscopes [9], scanning electron microscopes [10,11], laser scanners [12,13], borehole televiewers [14,15] etc., and internal structure measurements, which employ the computed tomography technique [16,17] and so on. As for the non-direct measurements, the physical and mechanical properties of target rock masses are firstly measured, and the blast-induced damage is then indirectly obtained by using those measured properties and calculating indexes representing rock damage. A diversity of physical and mechanical properties of rock masses can be used in calculating the blast-induced damage, among which stress-strain behavior [8,18], sonic wave velocities [19,20], electromagnetic wave response [21,22], acoustic emission characteristics [18,23,24], infrared radiation features [25,26], and electrical resistance values [27,28] etc. are all found commonly used in different occasions. Most of above measurement techniques, for example computed tomography and infrared cameras, are limited in laboratory tests so it is troublesome and impractical to apply them to evaluate the blast- induced damage in on-site operations. The measurement techniques widely adopted in on-site operations also have deficiencies of complicated operations, being time-consuming and costly. Nowadays, sonic tests, which are relatively simpler, cheaper and more timesaving, are the most commonly and pervasively used on-site measurement techniques for rock damage during excavation of high rock slopes.

The blast-induced damage correlates well with the peak particle velocity (PPV), and considerable effects have been made on predicting the blast-induced damage by developing a relationship between rock damage and the corresponding PPV [6], which helps reduce the time and cost for on-site measurement of the blast-induced damage. Holmberg and Persson [29] proposed an approach to predict damage zones for contour blasting based on an empirical equation relating rock damage and the PPV, and the empirical equation was later discretized by Hustrulid and Lu [30]. Subsequently, more adaptions and improvements were added onto the empirical equation for obtaining better performance in on-site applications [31,32,33]. Nowadays, soft computing methods are more and more popularly used in establishing empirical models for predicting rock damage [34,35,36], because those methods can address uncertainty and imprecision without knowing much about explicit theoretical expressions. Based on the relationship between rock damage and the corresponding PPV, the blast-induced damage can be detected by comparing monitored blasting vibration velocities against the critical PPV associated with a certain degree of rock damage. It should be noted that the near-field PPV and corresponding rock damage data are essential in establishing empirical predictive models. However, it is very difficult to obtain the near-field PPV data through the blasting vibration monitoring due to intense impacts and corresponding destructiveness in the vicinity of blastholes. In addition, the monitored blasting vibration data are influenced by not only rock damage but also many other factors such as measurement conditions, which easily leads to inexact prediction of the blast-induced damage.

Unlike blasting vibration velocities that are easily influenced by external conditions, natural frequencies of rock masses are intrinsic characteristics and relatively simple to obtain without knowing near-field vibration data. Based on commonly recorded blasting vibration data, natural frequencies of rock masses can be extracted by diverse methods, such as Fourier spectra [37,38], the power spectral density (PSD) [39,40], the transfer function [41,42], the frequency domain decomposition method [43,44], spectral ratios [45,46], and polarization analysis [40,47]. Researchers have developed a number of techniques to identify the location and degree of damage in structures using the change in the natural frequency in structural health monitoring [48,49,50]. Among those techniques of damage identification, the Bayesian approach that takes into account prior knowledge and posterior probabilities is one of the most appealing and prevailing techniques [51,52].

In the present study, a Bayesian approach to predict the blast-induced damage of high rock slopes using vibration and sonic data was proposed. A relationship between the blast-induced damage and the natural frequency was firstly developed. The blast-induced damage was obtained through sonic tests and the natural frequency was extracted by picking PSD peaks of blasting vibration monitoring data. Based on the relationship and available vibration and sonic data, a predictive model of the blast-induced damage was established by undertaking a Bayesian linear regression. By inputting the change rate in the natural frequency into the predictive model, the blast-induced damage for the next bench blasting can be predicted. The proposed Bayesian approach was finally adopted in the right bank rock slope at the Baihetan Hydropower Station. The results demonstrated that the proposed approach is feasible and efficient.

## 2. Relationship between Blast-Induced Damage and Natural Frequency

### 2.1. Blast-Induced Damage

We can define the change rate in the longitudinal wave velocity of rock masses before and after blasting as:

(1) $$\eta =\frac{C_{P}-\bar{C_{P}}}{C_{P}}=1-\frac{\bar{C_{P}}}{C_{P}}$$

where η is the change rate in the longitudinal wave velocity of rock masses, $C_{P}$ and $\bar{C_{P}}$ are the longitudinal wave velocities of rock masses before and after blasting, respectively.

According to construction technical specifications on rock foundation excavating engineering of hydraulic structures, rock masses are considered to be severely damaged when η exceeds 10% and the integrity of rock masses are critically destroyed. In engineering practice, the damage depth D is widely used to represent the blast-induced damage of rock masses and can be obtained by interpreting the change rate in the longitudinal wave velocity η at different depths from contour surfaces.

### 2.2. Natural Frequency of Rock Mass

**Figure 1:**
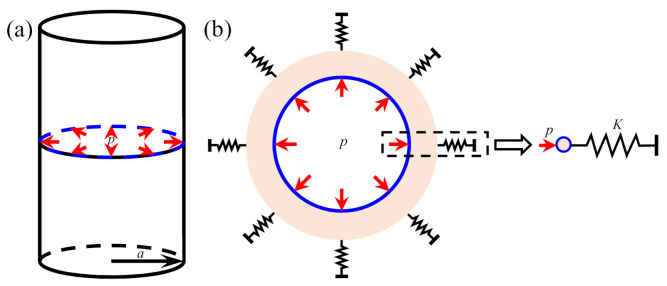
Simplified mechanical model for blasting of cylindrical charge: (**a**) Simplified model of infinite cylindrical charge in infinite rock masses; (**b**) Simplified plane and SDOF models.

As regards blasting of the cylindrical charge with infinite length in infinite rock masses, the cylindrical blasting source can be treated as a cylindrical cavity with a radius of a whose inner wall is acted upon the radial load p, as depicted in Figure 1a. Only considering radial motions of rock masses in the vicinity of the cylindrical cavity, the mechanical model shown in Figure 1a can be then simplified as a plane strain model as shown in Figure 1b. The simplified plane model can be further simplified into a single-degree-of-freedom (SDOF) model owing to that the infinite rock masses with the circular cavity are always symmetric regarding the circular cavity.

Assuming that rock masses are homogeneous, isotropic, and linear elastic media, the radial displacement of rock masses at distance r from the center of the blasting source can be obtained by:

(2) $$u_{r}=\frac{1+\upsilon }{E}\frac{a^{2}}{r}p$$

where υ and E are the Poisson’s ratio and the elastic modulus of rock masses, respectively.

Based on Equation (2), the equivalent radial stiffness *K* can be written as:

(3) $$K=\frac{2\pi ap}{u_{r}}=\frac{2\pi E}{1+\upsilon }\frac{r}{a}$$

The mass per unit length M of rock masses with the circular cavity follows as:

(4) $$M=\pi \left(r^{2}-a^{2}\right)\rho$$

where ρ is the mass density of rock masses.

Knowing the mass M and stiffness K, the natural frequency $f_{0}$ of rock masses can be identified by:

(5) $$f_{0}=\sqrt{\frac{K}{M}}=\sqrt{\frac{2}{1+\upsilon }}\frac{C_{P}}{a}\sqrt{\frac{ra}{r^{2}-a^{2}}}$$

When r≫a, the natural frequency $f_{0}$ is simplified as:

(6) $$f_{0}\approx \xi \frac{C_{P}}{a}\sqrt{\frac{a}{r}}$$

where ξ is a coefficient related to the properties of rock masses.

### 2.3. Relationship between Damage Depth and Natural Frequency

Though Equation (6) was developed based on several idealized assumptions, the assumptions can be proved valid in the vicinity of the blastholes [53], where the blast-induced damage is primarily triggered. Comparing Equation (1) with Equation (6), the damage depth D representing the blast-induced damage is roughly proportional to the change in the natural frequency $\Delta f_{0}$ of rock masses, as presented as Equation (7):

(7) $$D\propto \Delta f_{0}$$

Based on Equation (7) and available data of $\Delta f_{0}$, the damage depth D can be estimated by generating a predictive model describing the relationship between the damage depth and the change in natural frequency of rock masses.

## 3. Bayesian Approach to Predict Blast-Induced Damage

Excavation of high rock slopes follows the construction sequence from top to bottom. As the excavation advances, more and more on-site data from sonic tests and the blasting vibration monitoring at lower benches are progressively accumulated. In order to make the best of those accumulated data and update the predictive model in real time as new data are continually added, the Bayesian linear regression that can make full use of the prior knowledge and include the uncertainty of posterior parameters in predicted results [54] was adopted.

### 3.1. Bayesian Linear Regression

For a given dataset ${\left\{d_{i},y_{i}\right\}}_{i=1}^{N}$, where *N* is the number of data samples, $d_{i}\in R^{d}$ indicates the input variable, and $y_{i}\in R$ means the target value, the regression analysis aims at producing a predicted target value $y\left(d_{i};\omega \right)$ when the input variable $d_{i}$ is given. Equation (8) presents the expression of the linear regression:

(8) $$y\left(d_{t};\omega \right)=\sum_{i=1}^{n}\omega_{i}d_{t}^{i}+\omega_{0}$$

where $d_{t}^{i}$ indicates the *i*th element of the input variable $d_{t}$, and $\omega_{i}$ indicates the *i*th element of the weight vector ω.

Equation (8) can be also written as y=Φω, where $\Phi ={\left(\phi \left(d_{1}\right),\phi \left(d_{2}\right),\cdots ,\phi \left(d_{N}\right)\right)}^{T}$ and $y={\left(y_{1},y_{2},\cdots ,y_{N}\right)}^{T}$, and $\phi \left(d_{i}\right)$ is the basis function. Based on the Bayesian inference, the Bayesian linear regression is intended to get the solutions of the weight vector ω so as to establish the corresponding regression model.

The relationship between the predicted value $y\left(d_{i};\omega \right)$ and the target value $y_{i}$ follows:

(9) $$y_{i}=y\left(d_{i};\omega \right)+\theta_{i}$$

where $\theta_{i}$ is the noise and follows a Gaussian distribution, $\theta ∼N\left(0,\lambda^{-1}\right)$. As a result, the target value $y_{i}$ also follows a Gaussian distribution as written by Equation (10):

(10) $$p\left(y_{i}\left|\omega ,\lambda \right.\right)=N\left(y_{i}\left|y\left(d_{i};\omega \right)\right.,\lambda^{-1}\right)$$

For an input dataset $d={\left(d_{1},d_{2},\cdots ,d_{N}\right)}^{T}$, the likelihood of the target vector y is:

(11) $$p\left(y\left|\omega ,\lambda \right.\right)=\prod_{i=1}^{N}N\left(y_{i}\left|y\left(d_{i};\omega \right)\right.,\lambda^{-1}\right)={\left(\frac{\lambda }{2\pi }\right)}^{N/2}\exp\left\{-\frac{\lambda }{2}{\left\|y-\Phi \omega \right\|}^{2}\right\}$$

In order to avoid overfitting in the maximum-likelihood estimation and control the model complexity, a prior distribution is defined as:

(12) $$p\left(\omega \left|\zeta \right.\right)=\prod_{i=0}^{N}N\left(\omega_{i}\left|0\right.,\zeta^{-1}\right)$$

where ζ is the parameter controlling the distribution of $\omega_{i}$, and $p\left(\omega_{i}\left|\zeta \right.\right)=N\left(\omega_{i}\left|0\right.,\zeta^{-1}\right)$.

According to the Bayesian theorem, the posterior distribution of the weight vector ω is:

(13) $$p\left(\omega \left|y,\zeta ,\lambda \right.\right)=\frac{p\left(y\left|\omega ,\lambda \right.\right)p\left(\omega \left|\zeta \right.\right)}{p\left(y\left|\zeta ,\lambda \right.\right)}=N\left(\omega \left|\mu \right.,\Sigma \right)$$

where $\Sigma ={\left(\lambda \Phi^{T}\Phi +\zeta I\right)}^{-1}$ and $\mu =\lambda \Sigma \Phi^{T}y$ are the posterior variance and mean, respectively, and $p\left(y\left|\zeta ,\lambda \right.\right)=\int p\left(y\left|\omega ,\lambda \right.\right)p\left(\omega \left|\zeta \right.\right)d\omega$.

For a given test point $d_{*}$, the predicted distribution of the corresponding target value $y_{*}$ is:

(14) $$p\left(y_{*}\left|y\right.\right)=\int p\left(y_{*}\left|\omega ,\lambda \right.\right)p\left(\omega \left|y,\zeta ,\lambda \right.\right)p\left(\zeta ,\lambda \left|y\right.\right)d\omega d\zeta d\lambda$$

If $\zeta_{m}$ and $\lambda_{m}$ maximum $p\left(\zeta ,\lambda \left|y\right.\right)$, Equation (14) can be rewritten as:

(15) $$p\left(y_{*}\left|y\right.\right)=\int p\left(y_{*}\left|\omega ,\lambda_{m}\right.\right)p\left(\omega \left|y,\zeta_{m},\lambda_{m}\right.\right)d\omega =N\left(y_{*}\left|\mu_{*},\sigma_{*}^{2}\right.\right)$$

The predicted mean $\mu_{*}$ and variance $\sigma_{*}^{2}$ of the target value $y_{*}$ can be calculated by:

(16) $$\mu_{*}=\phi \left(d_{*}\right)\mu$$

(17) $$\sigma_{*}^{2}=\lambda_{m}^{-1}+\phi \left(d_{*}\right)\Sigma \phi {\left(d_{*}\right)}^{T}$$

### 3.2. Relationship between Damage Depth and Natural Frequency

**Figure 2:**
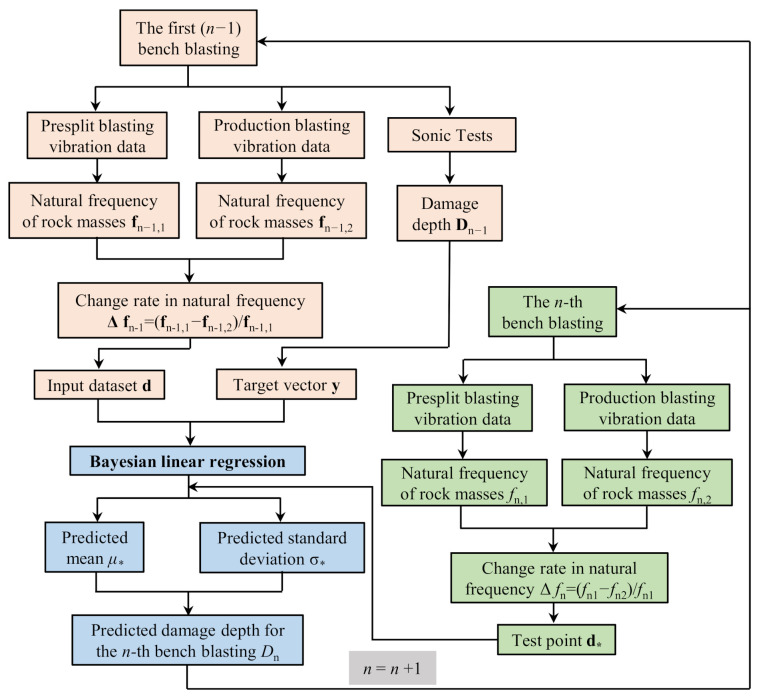
Procedure of Bayesian approach to predict blast-induced damage.

Procedures of the Bayesian approach to predict the blast-induced damage of high rock slopes using vibration and sonic data are illustrated in Figure 2. The procedures to predict the damage depth induced by the *n*-th bench blasting are divided into two major steps, namely establishing the predictive model and producing predicted results. For establishing the predictive model, blasting vibration monitoring and sonic test data from the first (*n*−1) bench blasting operations are firstly collected, among which the former data are used to identify natural frequencies of rock masses and the latter data are used to determine the damage depth. Then, the calculated natural frequencies $\Delta f_{n-1}$ are taken as the input dataset d and the damage depths $D_{n-1}$ are taken as the target vector y. Based on the input dataset d and the target vector y, the predictive model of the damage depth is finally developed by using the Bayesian linear regression. Since the predictive model has been developed, the predicted results of the damage depth for the *n-*th bench blasting are hence obtained just by inputting the change rate in the natural frequency $\Delta f_{n}$ before and after the *n-*th bench blasting into the predictive model. The above two major steps can be repeatedly conducted as the excavation of rock slopes advances. The procedures of the Bayesian approach have the advantages of introducing the prior information, considering the uncertainty, and improving the estimation as more data are collected.

## 4. Blasting and Measurement Operations at the Baihetan Hydropower Station

### 4.1. Engineering Background

**Figure 3:**
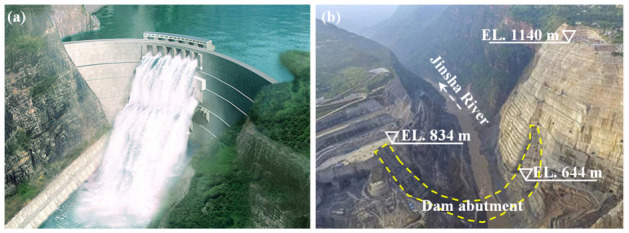
Baihetan Hydropower Station: (**a**) Double-curvature arch dam; (**b**) High rock slopes in dam abutment.

**Figure 4:**
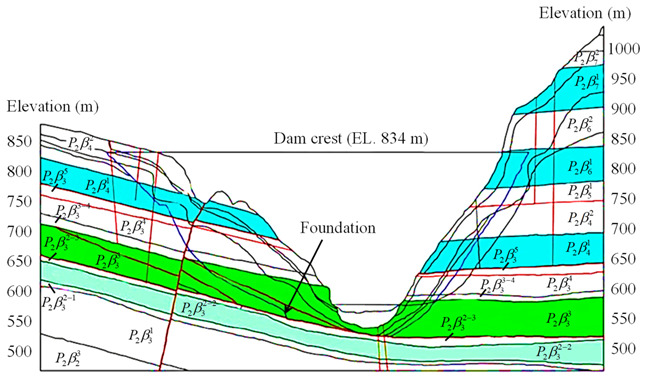
Typical geological profile of high rock slopes at Baihetan Hydropower Station.

The Baihetan Hydropower Station lies in an asymmetrical V-shaped canyon which is between Ningnan County in Sichuan Province and Qiaojia County in Yunnan Province, located in the lower course of the Jinsha River, southwest China. The station has a total installed capacity of 16,000 MW and the dam is a double-curvature arch dam with a maximum height of 289 m, as shown in Figure 3a. The natural slope angle of the left bank high rock slope is around 42° and that of the right bank high rock slope is around 65°. The heights of both the left and right bank high rock slopes in the dam abutment are between 200 m and 300 m, as shown in Figure 3b. The bedrocks of the high rock slopes in the dam abutment are mainly composed of Permian basalts $P_{2}\beta_{3}$~$P_{2}\beta_{6}$, and the typical geological profile of the high rock slopes is presented in Figure 4.

**Figure 5:**
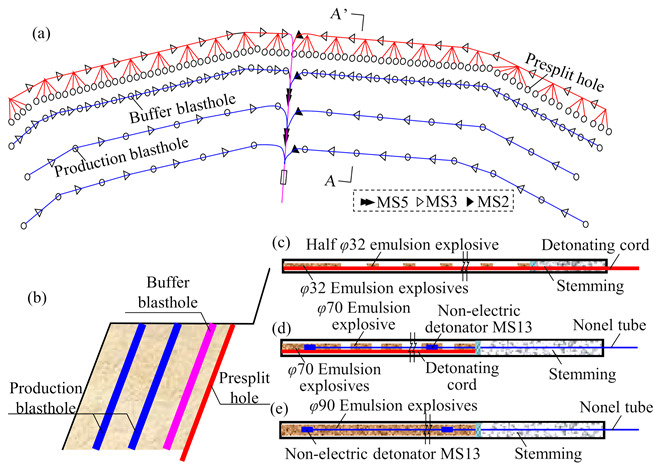
Typical blasting design of slope excavation between the EL. 794 m and the EL. 784 m: (**a**) Plane layout of blastholes; (**b**) Cross section of blastholes (A-A′); (**c**) Charge structure for presplit hole; (**d**) Charge structure for buffer blasthole; (**e**) Charge structure for production blasthole.

The excavation of the right bank high rock slope in the dam abutment was chose for study in this paper. Each bench height of the studied slope was designed to be 10 m. Blasting parameters used during the excavation of the slope were carefully determined based on a series of on-site experiments, and the detailed blasting parameters are summarized in Table 1. Initiation networks and charge structures commonly adopted in the blasting operations were basically similar. The typical initiation network of the blasting excavation between the EL. 794 m and the EL. 784 m and charge structures for different blastholes are shown in Figure 5.

### 4.2. Damage Depth Measurement

**Figure 6:**
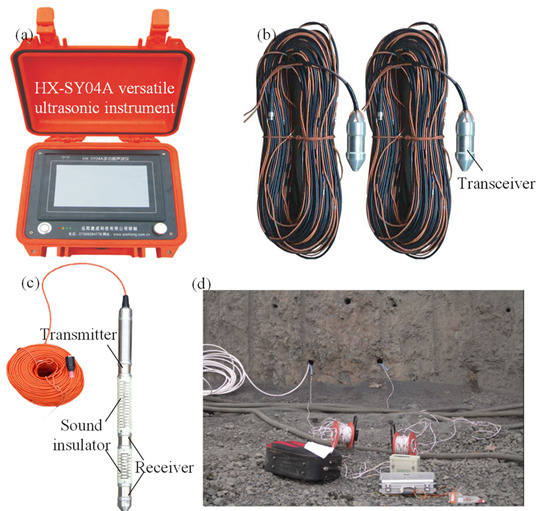
Sonic test system: (**a**) HX-SY04A versatile ultrasonic instrument; (**b**) Cross-hole transducer; (**c**) Single-hole transducer; (**d**) On-site sonic test.

Sonic tests before and after bench blasting were carried out to acquire the damage depth of each slope bench. The HX-SY04A sonic test system as presented in Figure 6, which is manufactured by Hunan Aocheng Technology Co., Ltd. (Shangsha, China), was employed to conduct sonic tests, and the sampling interval and measurement range in frequency bandwidth of the system are 0.1~499 μs and 10~200,000 Hz, respectively. The accuracy of sonic transit time of the system reaches 0.1 μs. Both the cross-hole and single-hole transducers were adopted in each sonic test, and the former is presented in Figure 6b and the latter is presented in Figure 6c.

In cross-hole sonic tests, the longitudinal wave velocity of rock masses is obtained as:

(18) $$C_{P}=\Delta d/\Delta t$$

where Δt is the time of the ultrasonic wave penetrating the rock masses between the two sonic test holes, and Δd is the minimum distance between the two sonic test holes.

In single-hole sonic tests, the longitudinal wave velocity of rock masses is obtained as:

(19) $$C_{P}=\Delta L/\left(t_{2}-t_{1}\right)$$

where $t_{1}$ and $t_{2}$ are the time of the ultrasonic wave travelling from the transmitter to the upper and lower receivers, respectively, and ΔL is the fixed length between the upper and lower receivers.

**Figure 7:**
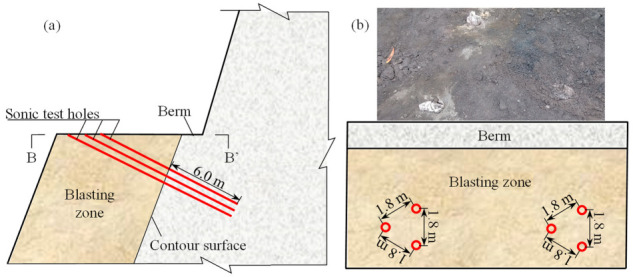
Layout of sonic test holes before bench blasting: (**a**) Cross section of sonic tests holes; (**b**) Plane view of sonic tests holes (B-B′).

**Figure 8:**
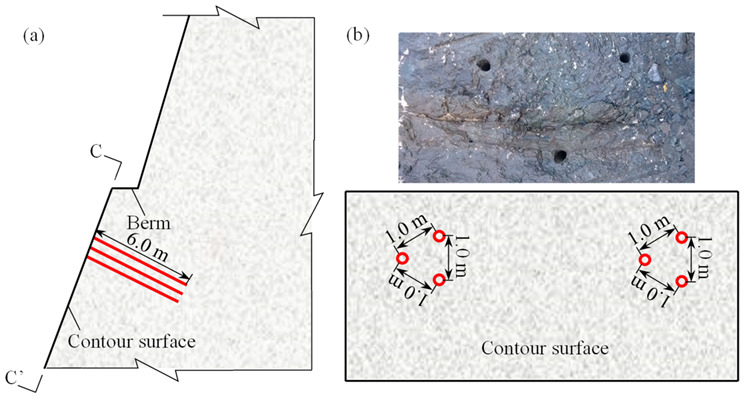
Layout of sonic test holes after bench blasting: (**a**) Cross section of sonic tests holes; (**b**) Section view of sonic tests holes (C-C′).

In order to guarantee the accuracy of the sonic tests, two groups of sonic test holes with the diameter of 90 mm were bored. According to the Chinese code for blasting safety monitoring of hydropower and water resources engineering and design requirement, all sonic test holes extended about 6 m from the contour surface. Each group of sonic test holes were arranged in form of an equilateral triangle, whose edge lengths in the berm surface and the contour surface are about 1.8 m and 1.0 m, respectively. The typical layout of the sonic test holes before bench blasting is depicted in Figure 7. Before bench blasting, total 12 sonic tests including six cross-hole sonic tests and six single-hole sonic tests were performed to gain the longitudinal wave velocities of the remaining rock masses before bench blasting. As the bench blasting was done, the layout of the remaining sonic test holes turned to be in the form as shown in Figure 8. After removing the stemmed rock debris in the sonic test holes, another 12 sonic tests including 6 cross-hole sonic tests and 6 single-hole sonic tests were again performed to gain the longitudinal wave velocities of the remaining rock masses after bench blasting.

**Figure 9:**
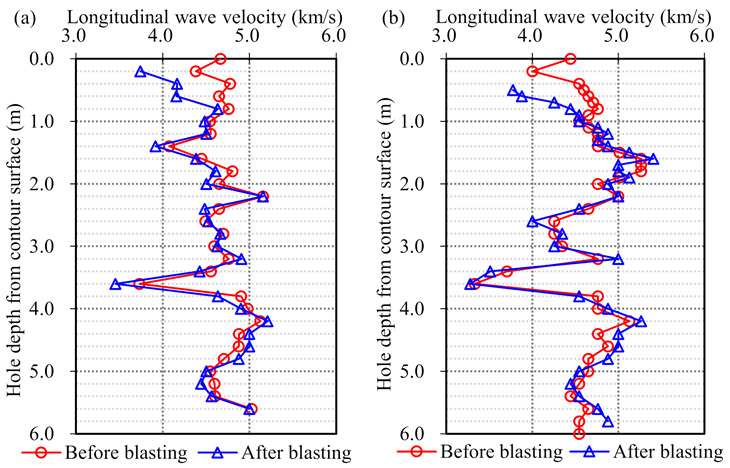
Typical results of sonic tests between the EL. 794 m and the EL. 784 m: (**a**) Typical results of cross-hole sonic test; (**b**) Typical results of single-hole sonic test.

Typical results of the sonic tests before and after bench blasting are plotted in Figure 9, which show the change in longitudinal wave velocities of rock masses. According to the results of the sonic tests and Equation (1), the damage depth for each slope bench was determined by averaging the results of both cross-hole and single-hole sonic tests.

### 4.3. Blasting Vibration Monitoring

**Figure 10:**
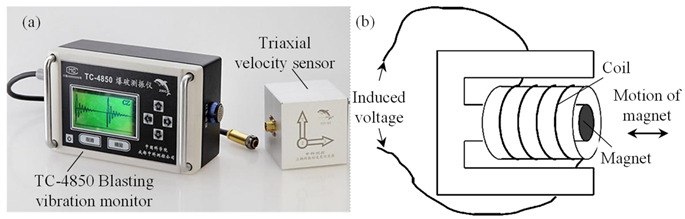
Blasting vibration monitoring system: (**a**) TC-4850 intelligent monitor and triaxial velocity sensor; (**b**) Internal component of velocity sensor.

The blasting vibration monitoring during the bench blasting was implemented to further extract the natural frequencies of rock masses. The TC-4850 blasting vibration monitoring system as shown in Figure 10a, which is manufactured by Chengdu Zhongke Measurement and Control Co., Ltd. (Chengdu, China), was used in recording the blasting vibration and it is composed of the TC-4850 intelligent monitor and the matched triaxial velocity sensor. The internal component of the velocity sensor comprises a coil and a suspended magnet, as depicted in Figure 10b. The measurement ranges in the velocity and the frequency of the blasting vibration monitoring system are 0.001~35.0 cm/s and 5~300 Hz, respectively. The velocity resolution of the system is 0.01 cm/s.

**Figure 11:**
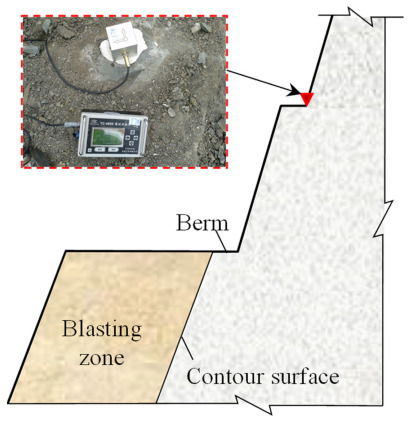
Typical layout of blasting vibration monitoring system.

**Figure 12:**
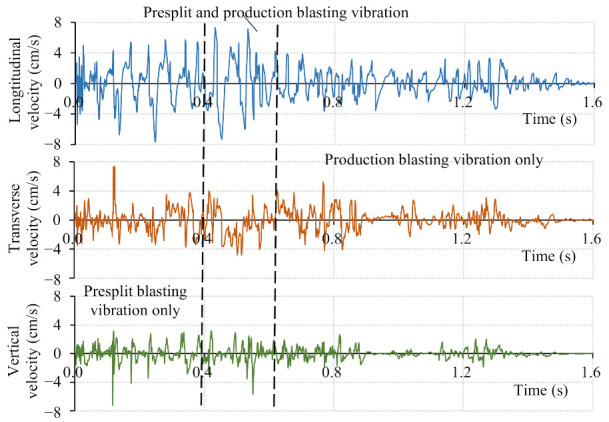
Typical blasting vibration waveforms of monitoring point at EL. 804 m.

Considering the reliability of the recorded vibration data and the safety of the monitoring system, the blasting vibration monitoring system was arranged at the toe of the upper slope bench, as shown in Figure 11. The recorded blasting vibration waveforms of the monitoring point mounted at the EL. 804 m are typically presented in Figure 12. According to the initiation network shown in Figure 5a, the blasting vibration waveforms shown in Figure 12 can be divided into three segments: the presplit blasting vibration waveform, the production blasting vibration waveform, and the superposition of the presplit and production blasting vibration waveform. The presplit blasting vibration waveform was used to extract the natural frequency of rock masses between the monitoring point and the blasting zone before bench blasting, and the production blasting vibration waveform was used to extract the natural frequency of rock masses after bench blasting. The change in the natural frequency of rock masses was then adopted to predict the damage depth of the rock masses adjacent to the blasting zone.

The power spectral density (PSD), which describes how the power of a signal or time series is distributed over the frequency, of the recorded blasting vibration data was used to extract the natural frequencies of rock masses before and after bench blasting. The PSD of a signal $x\left(t\right)$ is simply defined as:

(20) $$S_{xx}\left(f\right)=\underset{T\to \infty }{\lim}\frac{{\left|{\tilde{x}}_{T}\left(f\right)\right|}^{2}}{T}$$

where $x_{T}\left(t\right)=x\left(t\right)\omega_{T}\left(t\right)$ and $\omega_{T}\left(t\right)$ is the unity within the arbitrary period and zero elsewhere, and ${\left|{\tilde{x}}_{T}\left(f\right)\right|}^{2}=\int_{-\infty }^{\infty }\left[\int_{-\infty }^{\infty }x_{T}^{*}\left(t-\tau \right)x_{T}\left(t\right)dt\right]e^{-i2\pi f\tau }d\tau$ is the Fourier transform of the time convolution of $x_{T}^{*}\left(-t\right)$ and $x_{T}\left(t\right)$.

Considering a window of −N≤n≤N with the signal sampled at discrete times $x_{n}=x\left(n\Delta t\right)$ for a total measurement period $T=\left(2n+1\right)\Delta t$, the PSD defined as Equation (20) can be generalized to discrete time variables $x_{n}$ as:

(21) $$S_{xx}\left(f\right)=\underset{N\to \infty }{\lim}\frac{{\left(\Delta t\right)}^{2}}{T}{\left|\sum_{n=-N}^{N}x_{n}e^{-i2\pi fn\Delta t}\right|}^{2}$$

**Figure 13:**
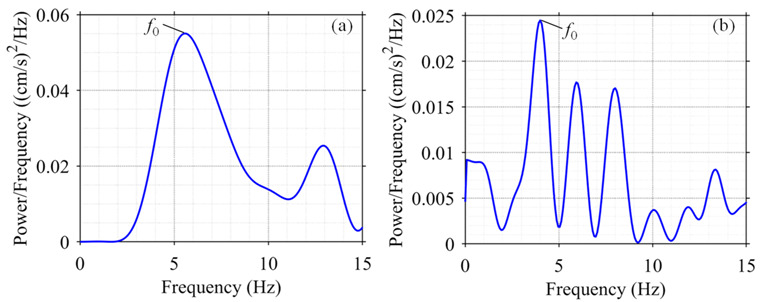
Typical PSD illustrations of longitudinal velocities for monitoring point at EL. 804 m: (**a**) PSD of presplit blasting vibration; (**b**) PSD of production blasting vibration.

According to the previous derivation of the relationship between the damage depth and the natural frequency, the radial motion of rock masses are closely related to the blast- induced damage and hence longitudinal blasting vibration velocities were employed to extract the natural frequencies of rock masses. The typical PSD illustrations of the longitudinal velocities for the monitoring point at EL. 804 m are plotted in Figure 13. The frequencies corresponding to the peaks in the PSD reveal the resonance frequencies that can be considered to be equal to the natural frequencies of the rock masses. The first resonance frequency was selected for calculating the change in the natural frequency of rock masses.

## 5. Results and Discussion

### 5.1. Damage Depth and Change in Natural Frequency

**Figure 14:**
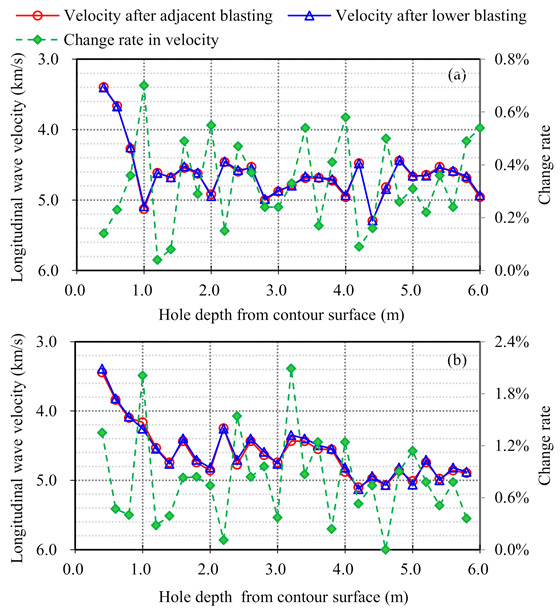
Typical results of repeated sonic tests of the same remaining rock masses for different bench blasting: (**a**) Cross-hole sonic test; (**b**) Single-hole sonic test.

It is important to clarify the impact of the lower bench blasting on the damage state of the upper remaining rock masses, because the blasting vibration monitoring system for the current bench blasting was arranged at the toe of the upper slope bench. Therefore, repeated sonic tests of the same remaining rock masses were separately conducted after the adjacent and lower bench blasting. The typical results of the repeated sonic tests and their differences are presented in Figure 14. The results show that the differences between the longitudinal wave velocities of the same remaining rock masses after the adjacent and lower bench blasting are all lower than 2.0% and most of them are below 1.0%. The differences are rather small so that the lower bench blasting can be considered to have hardly any impact on the damage state of the upper remaining rock masses. As a result, the change in the natural frequency extracted from the blasting vibration data recorded at the upper bench toe can reflect the change of damage state induced by the current bench blasting.

**Figure 15:**
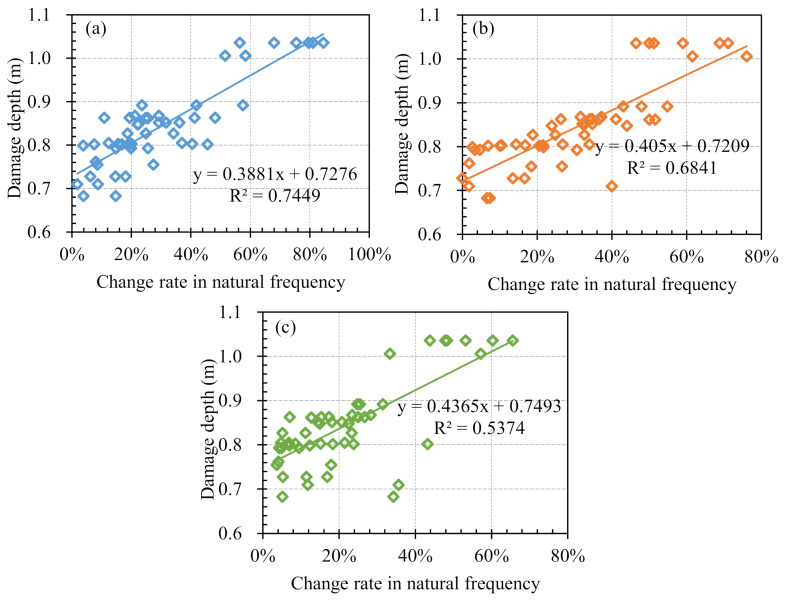
Scatter plots between damage depth and change rate in natural frequency: (**a**) Longitudinal vibration; (**b**) Transverse vibration; (**c**) Vertical vibration.

Data of sonic tests and blasting vibration monitoring were collected from total 19 bench blasting operations, and total 52 sets of data pairs comprising the damage depth and the change in the natural frequency were obtained through the collected data, Equation (1) and Equation (21). The scatter plots shown in Figure 15 reveal the relationship between the damage depth and the change rate in the natural frequency. According to the scatter plots and corresponding linear fitting equations, the damage depth is found to be proportional to the change rate in the natural frequency, which conforms to the relationship expressed by Equation (7). In addition, the correlation coefficient *R* of the linear fitting equation calculated through the longitudinal velocities is the largest, which verifies the reliability and superiority of using longitudinal vibration data instead of transverse and vertical vibration data.

### 5.2. Predicted Results of Damage Depth

As the excavation of high rock slopes advances, more and more on-site data coming from sonic tests and the blasting vibration monitoring were collected, and those increasing data were successively used in the progressive procedures presented in Figure 2 to update the developed predictive model continuously and improve the prediction reliability.

**Figure 16:**
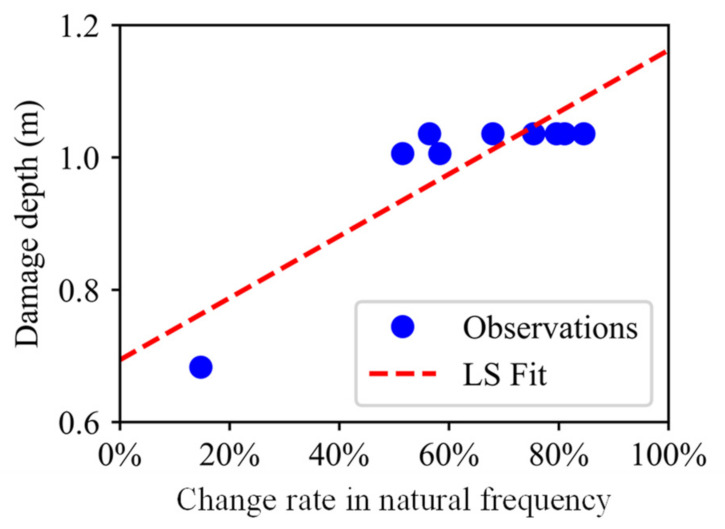
Scatter plot and the fitting line between damage depth and change rate in natural frequency (first two bench blasting operations).

For the first two bench blasting operations, there are total 9 sets of data pairs relating the damage depth to the change rate in the natural frequency, and the corresponding scatter plot and the fitting line derived by the least square (LS) method are both shown in Figure 16. In the Bayesian approach, the linear relationship between the damage depth and the change rate in the natural frequency is represented as:

(22) $$D=k_{0}\Delta \tilde{f}+k_{1}+\theta$$

where $k_{0}$ and $k_{1}$ are the slope and intercept of the fitting line, respectively, and θ indicates the noise.

**Figure 17:**
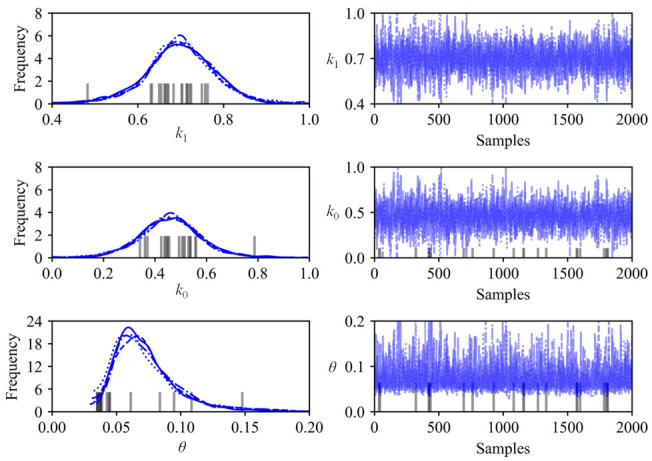
Posterior distribution and individual samples (first two bench blasting operations).

**Figure 18:**
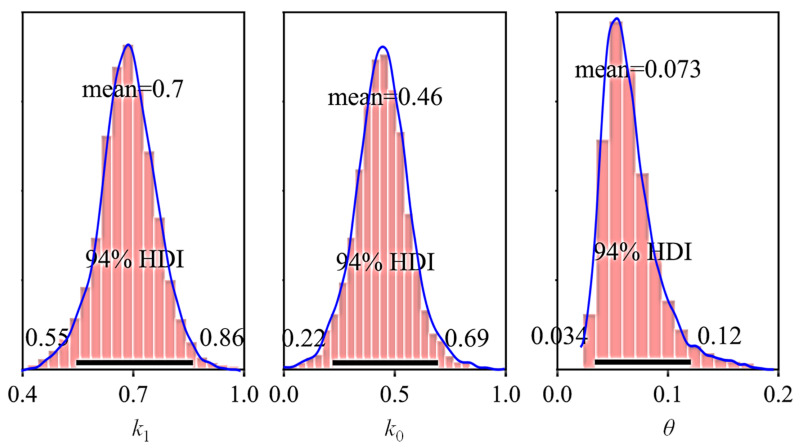
Posterior distribution with mean value and HDI of 94% (first two bench blasting operations).

The slope and intercept of the fitting line obtained by LS method were selected as the initial values for the parameters $k_{0}$ and $k_{1}$, and then the Markov chain Monte Carlo (MCMC) algorithm was used to draw 2000 posterior samples. The posterior distribution of the parameters ($k_{0}$, $k_{1}$, and θ) and the corresponding individual samples are drawn in Figure 17. The posterior distributions of the above parameters with the mean value and the highest density interval (HDI) of 94% were further specifically presented in Figure 18.

**Figure 19:**
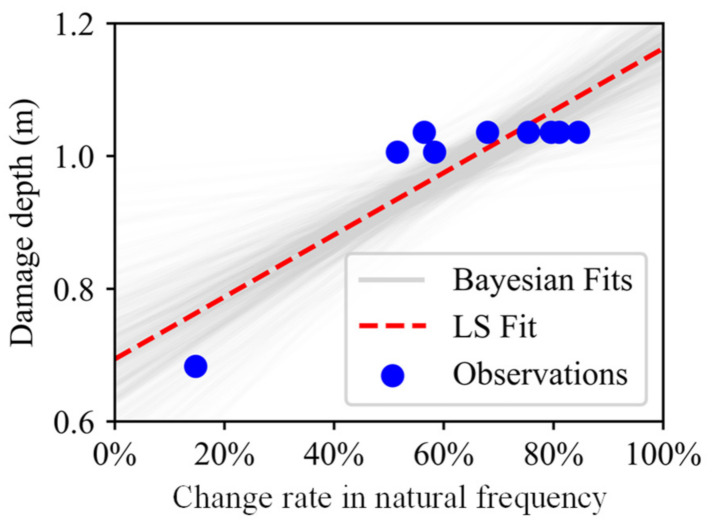
Posterior predictive plots of Bayesian fits (first two bench blasting operations).

The posterior predictive regression lines marked as Bayesian fits in Figure 19 were obtained by taking multiple samples from the posteriors of the intercept and slope and plotting a regression line for each of them. The estimated regression lines of the Bayesian fits are similar to the regression line of LS fit, but there exists uncertainty in Bayesian estimates that is expressed by the variability of the regression lines of the Bayesian fits. Therefore, the predictive model of the damage depth is represented by the posterior predictive lines of the Bayesian fits.

**Figure 20:**
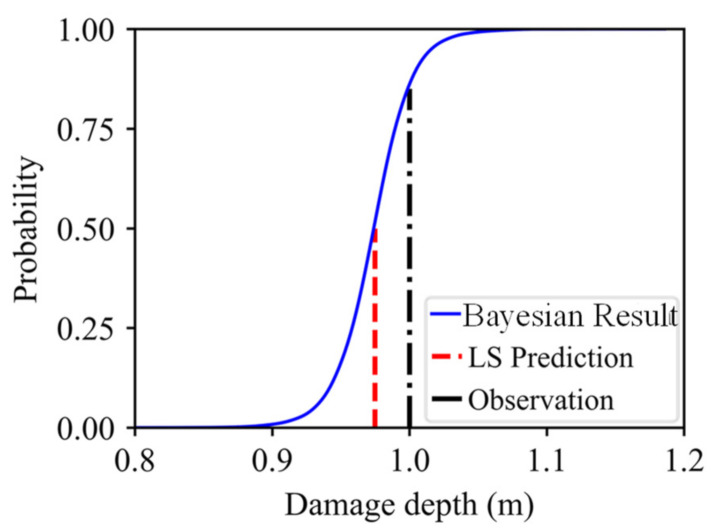
Bayesian predicted result of damage depth for the third bench blasting (cumulative distribution function curve).

Since the predictive model of the damage depth was determined through the Bayesian approach, the damage depth for the third bench blasting could be predicted by inputting the change rate in the natural frequency of rock masses for the third bench blasting. Figure 20 shows the predicted result of the damage depth for the third bench blasting. From the cumulative distribution function curve of the predicted damage depth, it can be seen that the result of the LS prediction is far away from the on-site observation, while the result of Bayesian prediction covers the result of the LS prediction and the on-site observation. The Bayesian predicted result indicates the probability of that the damage depth induced by the third bench blasting is lower than the on-site observation is about 0.85.

**Figure 21:**
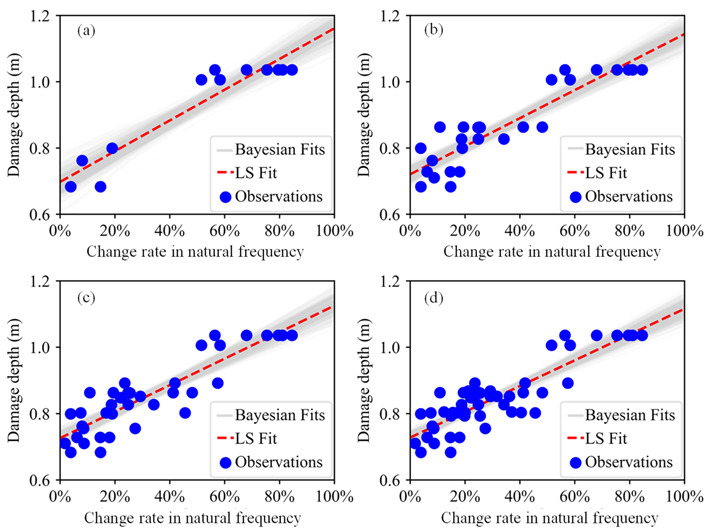
Typical Bayesian predictive models: (**a**) The 4th bench blasting; (**b**) The 9th bench blasting; (**c**) The 14th bench blasting; (**d**) The 19th bench blasting.

**Figure 22:**
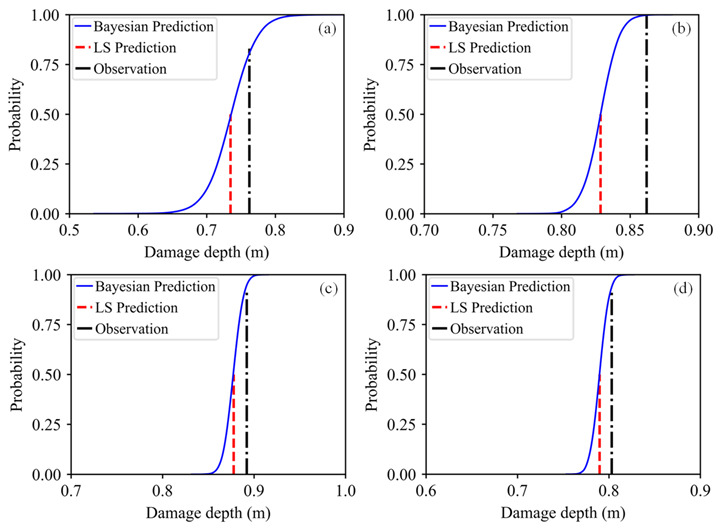
Typical Bayesian predicted results: (**a**) The 4th bench blasting; (**b**) The 9th bench blasting; (**c**) The 14th bench blasting; (**d**) The 19th bench blasting.

The complete procedures of the Bayesian approach to predict the damage depth for the third bench blasting are illustrated in Figure 16, Figure 17, Figure 18, Figure 19 and Figure 20, and the procedures could be divided into two major steps, firstly developing a Bayesian predictive model of the damage depth using collected on-site data of the first two bench blasting operations and then predicting the damage depth by inputting the change rate in the natural frequency for the third bench blasting into the predictive model. Using the Bayesian approach, the predictive model for each bench blasting operation can be developed and the prediction of the damage depth induced by the next bench blasting can be obtained. The typical predictive models and predicted results of the damage depth for other bench blasting operations are presented in Figure 21 and Figure 22, respectively.

As shown in Figure 21, the Bayesian predictive model for each bench blasting is dynamically adjusted and gradually becomes steady with the increasing input data. Compared with the single regression line of the LS fit, the regression zone of the Bayesian fits is composed of a series of similar single regression lines and expresses the estimation uncertainty by the variability of those regression lines. The Bayesian predicted results shown in Figure 22 show that the differences between the LS predicted results and the on-site observations are large, while the Bayesian predicted results cover both of them. The cumulative distribution function curve of the predicted damage depth presented in Figure 22a indicates the probability of that the damage depth induced by the 4th bench blasting is below the corresponding on-site observations is about 0.86. The cumulative distribution function curve presented in Figure 22b indicates the probability of that the damage depth induced by the 9th bench blasting is below the corresponding on-site observations is about 0.99. The cumulative distribution function curves presented in Figure 22c,d indicate the probabilities of that the damage depths induced by the 14th and 19th bench blasting are below the corresponding on-site observations are about 0.92. All the probabilities of predicted results being below corresponding observations are all above 0.85.

The proposed Bayesian approach uses only parts of the blasting vibration monitoring and sonic test data that are originally required for controlling the vibration and damage of the remaining rock masses, and no additional data are further required. Developing the predictive model in the Bayesian approach using the natural frequency instead of the PPV helps reduce the measurement work and the prediction deviation, because at least five blasting vibration monitoring points along a line are demanded in exploring the blasting vibration attenuation law that is used to predict the PPV while just one blasting vibration monitoring point at the upper bench toe is enough for extracting the natural frequency of rock masses. By using the Bayesian linear regression, the new blasting vibration monitoring and sonic test data can be added into the input dataset to update the predictive model. Furthermore, the Bayesian predicted result is not the distinct point but a distribution containing some statistical characteristics that describe the damage state more appropriately and scientifically. Further studies will be considered to integrate the statistical characteristics into the current description and codes of the damage and vibration control.

## 6. Conclusions

Considering the benefits that the natural frequencies of rock masses are intrinsic characteristics and relatively simple to obtain without knowing the near-field vibration data, a relationship between the blast-induced damage and natural frequency of rock masses was firstly developed. The damage depth representing the blast-induced damage is proportional to the change in the natural frequency. The blast-induced damage was obtained through sonic tests and the natural frequencies were extracted by picking PSD peaks of blasting vibration monitoring data. Based on the developed relationship and available vibration and sonic data, a Bayesian approach was then proposed to predict the blast-induced damage of high rock slopes using vibration and sonic data. The procedures of the Bayesian approach are divided into two major steps, namely establishing the predictive model and producing the predicted results. The Bayesian predictive models of the damage depth were firstly developed by undertaking the Bayesian linear regression. There exists uncertainty in the Bayesian estimates that was expressed by the variability of the regression lines of the Bayesian fits. The damage depth for the next bench blasting could be predicted by inputting the change rate in the natural frequency of rock masses to the predictive models. Finally, the Bayesian approach was applied in the Baihetan Hydropower Station, and the probabilities of predicted results being below corresponding observations are all above 0.85. The proposed Bayesian approach that makes the best of numerous monitoring data and includes the uncertainty in the predicted results is practical and efficient. This study focuses on predicting the blast-induced damage of high rock slopes and the presented case study at Baihetan Hydropower Station provides a reference for similar projects.

## Figures and Tables

**Table 1:** Detailed blasting parameters.

Blasthole	Blasthole Parameter				Charge Parameter		
	Diameter (mm)	Length (m)	Spacing (m)	Burden (m)	Diameter (mm)	Stemming (m)	Weight per Blasthole (kg)
Presplit hole	90	10.4~11.2	0.8	/	32	1.0	5.2~7.4
Buffer blasthole	105	10.4~11.2	1.9	1.4	70	3.0	34~42
Production blasthole	105	9.8~12.4	5.0	3.0	90	3.0	50~64

## Data Availability

The data presented in this study is available on request from the corresponding author.
